# Publisher Correction: Application of full-genome analysis to diagnose rare monogenic disorders

**DOI:** 10.1038/s41525-021-00251-3

**Published:** 2021-10-12

**Authors:** Joseph T. Shieh, Monica Penon-Portmann, Karen H. Y. Wong, Michal Levy-Sakin, Michelle Verghese, Anne Slavotinek, Renata C. Gallagher, Bryce A. Mendelsohn, Jessica Tenney, Daniah Beleford, Hazel Perry, Stephen K. Chow, Andrew G. Sharo, Steven E. Brenner, Zhongxia Qi, Jingwei Yu, Ophir D. Klein, David Martin, Pui-Yan Kwok, Dario Boffelli

**Affiliations:** 1 Institute for Human Genetics, University of California San Francisco, San Francisco, CA USA; 2 Division of Medical Genetics, Pediatrics, Benioff Children’s Hospital, University of California San Francisco, San Francisco, CA USA; 3 Cardiovascular Research Institute, University of California San Francisco, San Francisco, CA USA; 4 Biophysics Graduate Group, University of California Berkeley, Berkeley, CA USA; 5 Department of Plant and Microbial Biology, University of California Berkeley, Berkeley, CA USA; 6 Department of Laboratory Medicine, University of California San Francisco, San Francisco, CA USA; 7 Craniofacial Biology and Department of Orofacial Sciences, University of California San Francisco, San Francisco, CA USA; 8 Children’s Hospital Oakland Research Institute, Benioff Children’s Hospital Oakland, University of California San Francisco, Oakland, CA USA; 9 Department of Dermatology, University of California San Francisco, San Francisco, CA USA

**Keywords:** Paediatrics, Medical genetics

Correction to: *npj Genomic Medicine* 10.1038/s41525-021-00241-5, published online 23 September 2021

“The original version of the published Article contained additional text that was inadvertently inserted in the first line of the abstract. The following text was removed from the abstract to improve clarity: ‘enhancer and narrows the diagnostic interval’. The HTML and PDF versions of the Article have been corrected.”

