# Supplementary material for: Application of full-genome analysis to diagnose rare monogenic disorders
Source: NPJ Genom Med. 2021 Sep 23;6:77. doi: 10.1038/s41525-021-00241-5 (PMC8460793; doi:10.1038/s41525-021-00241-5)
Supplement: Supplementary file 1 — Supplementary Information pdf [file 41525_2021_241_MOESM1_ESM.pdf]

Supplementary information for

Application of Full Genome Analysis to Diagnose Rare Monogenic Disorders

Authors: Joseph T. Shieh[1,2,*], Monica Penon-Portmann[1,2,*], Karen H.Y. Wong[3,*], Michal Levy-Sakin[3], Michelle Verghese[3], Anne Slavotinek[1,2], Renata Gallagher[1,2], Bryce A. Mendelsohn[2], Jessica Tenney[2], Daniah Beleford[2], Hazel Perry[2], Stephen K. Chow[3], Andrew G. Sharo[4], Steven E. Brenner[5], Zhongxia Qi[6], Jingwei Yu[6], Ophir D. Klein[1,2,7], David Martin[8], Pui-Yan Kwok[1,3,9], Dario Boffelli[8]

Corresponding author:
Joseph Shieh, MD PhD
Joseph.shieh2@ucsf.edu

This file includes:

Supplementary Figure 1. Duplicated intronic region in NHEJ1 and GeneHancer

Supplementary Figure 2. Karyotype and ideogram of genomic rearrangement, 46,XY,t(1;9)(p32.3,p21)

Supplementary Figure 3. Duplication of 7q11.23, subsequently translocated to 2q37.3 in an inverted orientation

Supplementary Figure 4. De novo heterozygous 1480bp deletion disrupting *WAC*

Supplementary Figure 5. Flowchart depicting the automated variant interpretation pipeline

Supplementary Table 1. Pipeline performance.

Supplementary Table 2. Additional thirty cases tested with FGA and candidates identified.

Supplementary Table 3. Comparison of inter-chromosomal events between short-read WGS CNV and genome assembly technologies for proband 0703 with a 46,XY,t(1;9)(p32.3;p21) rearrangement.

Supplementary Table 4. Comparison of inter-chromosomal events between short-read WGS CNV and genome assembly technologies for a proband 4603 with a complex rearrangement. Duplication of 7q11.23, subsequently translocated to 2q37.3 in an inverted orientation [der(2)t(2;7)(q37.3; q11.23)dup(7)(q11.23;q11.23)]

Supplementary Table 5. Comparison of deletion calls between short-read WGS CNV and genome assembly technologies. Table includes calls for proband 5103 with 36 kb *TANGO2* deletion.

Supplementary Table 6. Comparison of deletion calls between short-read WGS CNV caller and genome assembly technologies. Table includes calls for proband 4203 with 1480 bp *WAC* deletion.

Supplementary Table 7. Comparison of deletion calls between short-read WGS CNV caller and genome assembly technologies. Table includes calls for proband 4803 with 5000 bp deletion disrupting *USP34*.

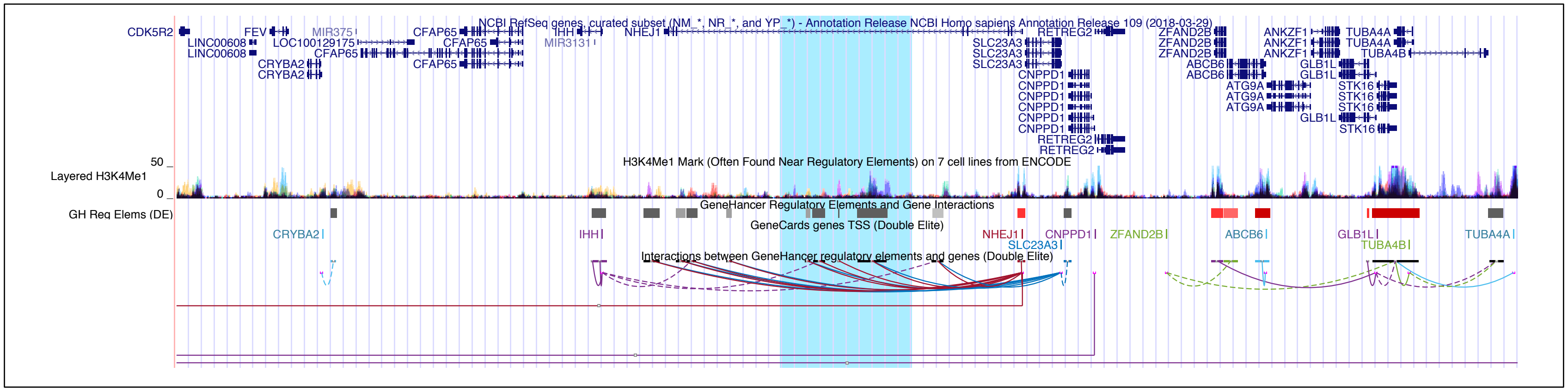

Supplementary Figure 1. Duplicated region in *NHEJ1* and GeneHancer. View from UCSC Genome Browser of the duplicated region from non-coding variant (highlighted in light-blue, chr2: 219,102,933-219,134,970, GRCh38) and surrounding genes. The tracks displayed demonstrate how the duplication occurs in an intronic region that affects regulatory elements. The H3K4Me1 Mark shows where modification of histone proteins is highly suggestive of an enhancer. The GeneHancer track shows associations between regulatory elements (grey bars) and their target genes, in this case, *IHH* and *NHEJ1* (purple and red lines).

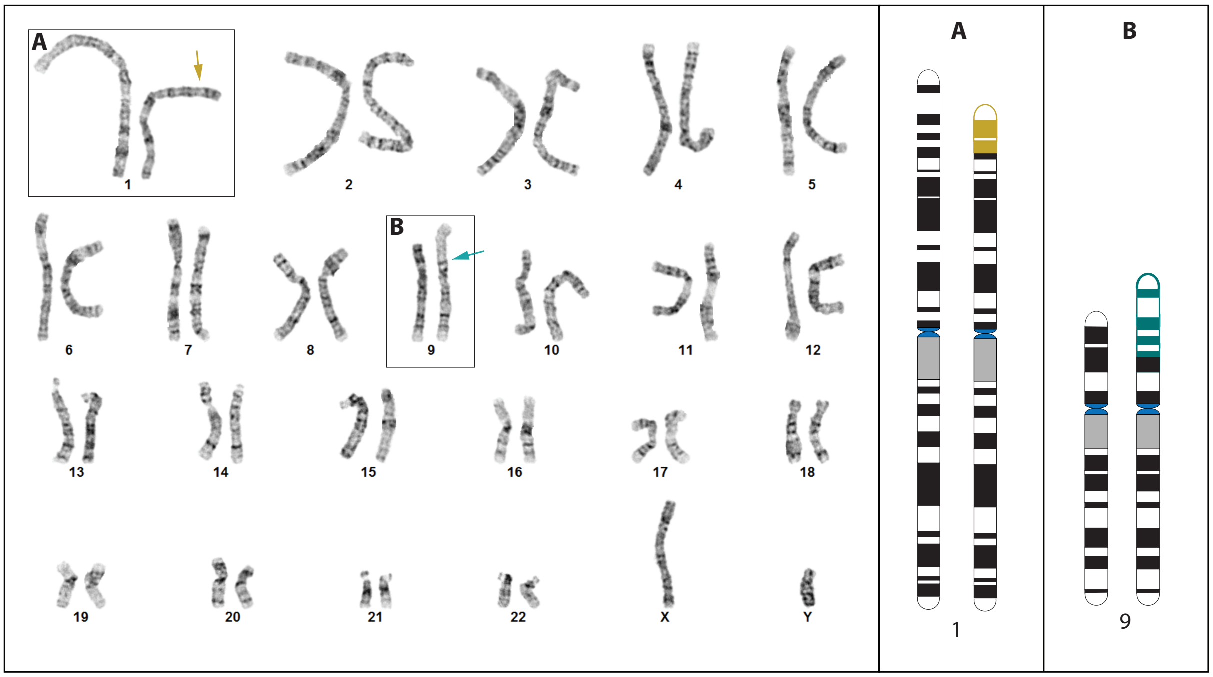

Supplementary Figure 2. Karyotype and ideogram of genomic rearrangement, 46,XY,t(1;9)(p32.3,p21). Left panel: Karyotype validating the FGA finding with arrows pointing to breakpoints**. Right panel:** Ideogram of the translocation.

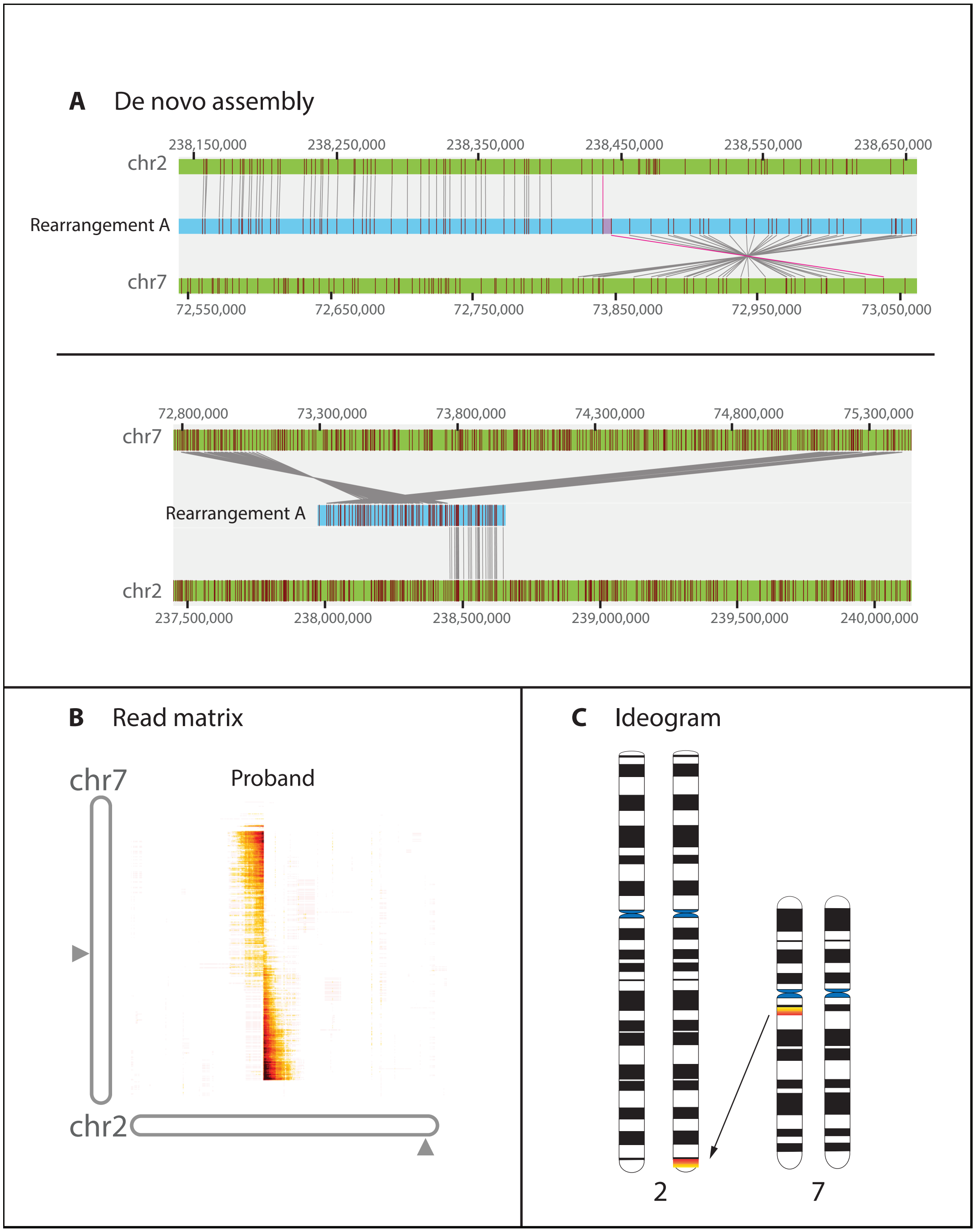

Supplementary Figure 3. Duplication of 7q11.23, subsequently translocated to 2q37.3 in an inverted orientation, case 4603 [der(2)t(2;7)(q37.3; q11.23)dup(7)(q11.23;q11.23)]. **Panel A, top**: a *de novo* scaffold (rearrangement A in light blue) with the translocated region that aligns to chromosome 2 reference (in green) and chromosome 7 reference (in green) in an inverted orientation. **Panel A, bottom**: a *de novo* scaffold (rearrangement A in light blue) of the segmental duplication in 7q11. **Panel B:** matrix view with unexpected barcode overlap between chr2:238,439,963-238,439,968 and chr7:72,774,109-73,100,000 (genome version GRCh38). **Panel C:** An ideogram of the rearrangement. The yellow-orange gradient depicts the inverse orientation. This is a maternally inherited rearrangement.

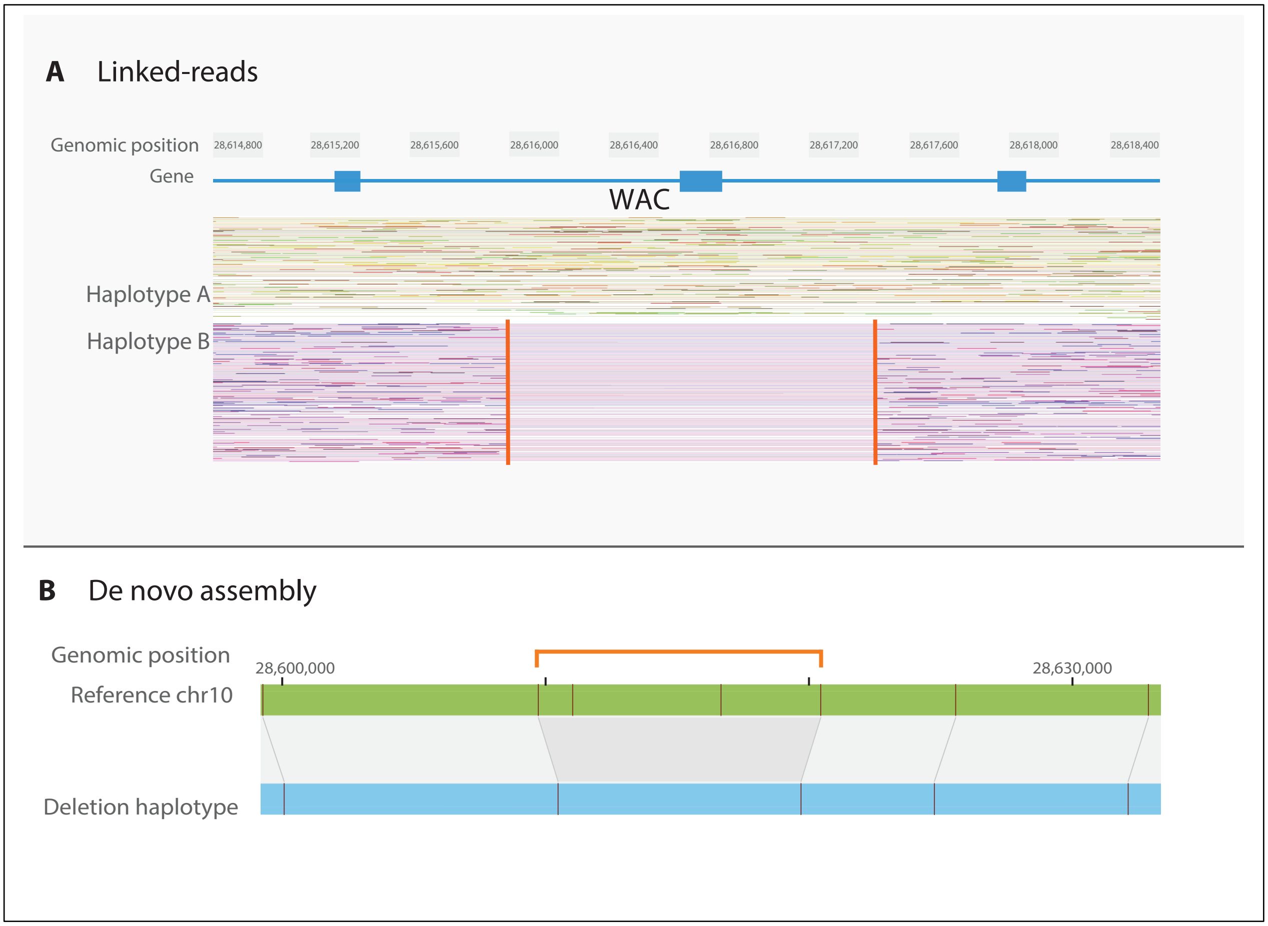

Supplementary Figure 4. ***De novo*** **heterozygous 1.5kb deletion disrupting** ***WAC*** (chr10:28,615,989-28,617,469, genome version GRCh38, case 4203). **Panel A:** Deletion is seen by drop in coverage in haplotype B (orange lines). **Panel B:** De novo assembly (light blue) demonstrates missing sequence labels with respect to reference (green). The orange bracket and gray triangle depict the deleted region.

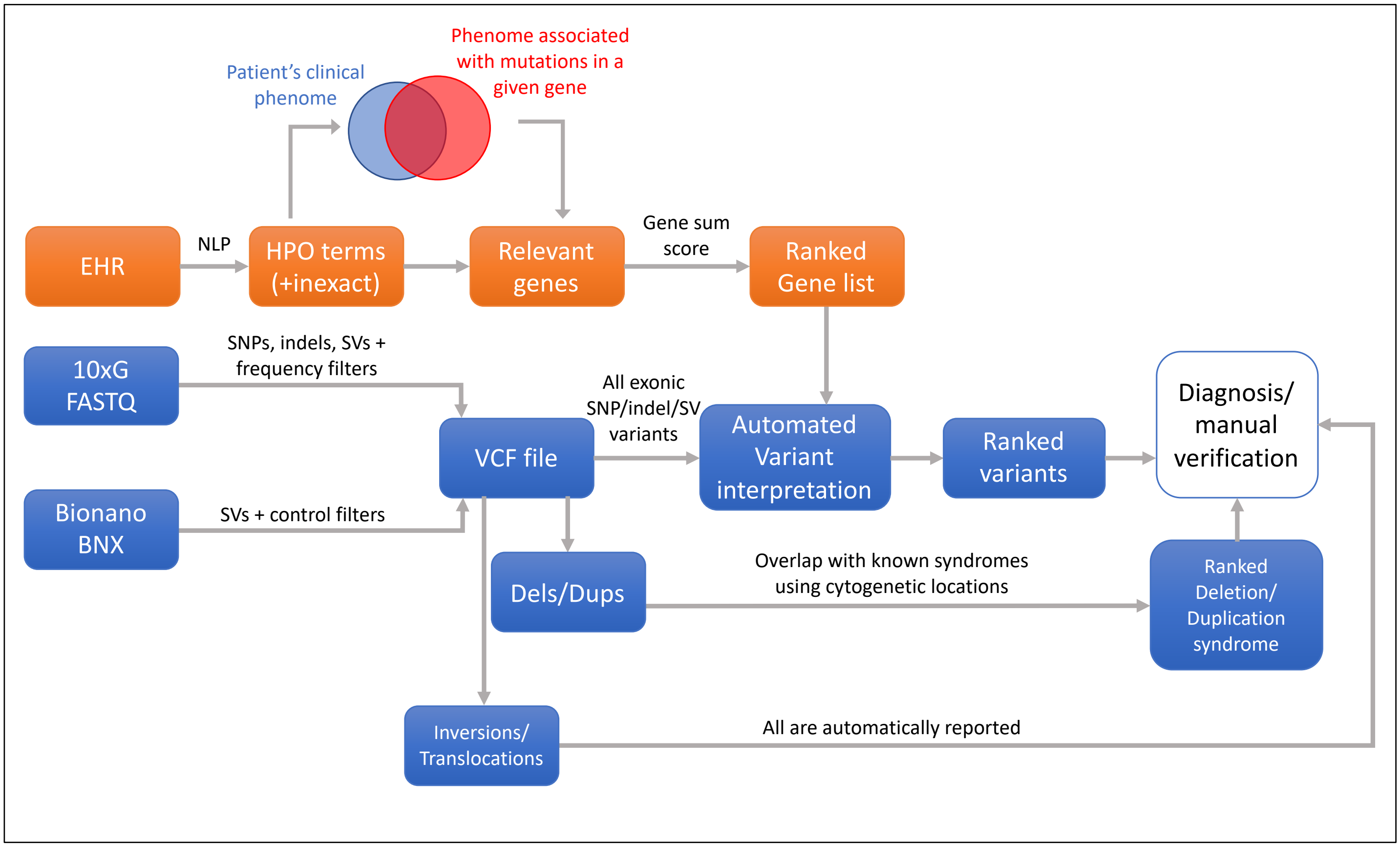

Supplementary Figure 5. Flowchart depicting the automated variant interpretation pipeline. The pipeline incorporates, EHR derived HPO terms and multiple types of genomic variants. In orange, the electronic health records from the probands were exported as JSON format. Unstructured clinical notes were translated into HPO terms using clinical natural language processing tool. Related HPO hierarchical terms separated by 1 degree were also included as part of the clinical phenome, which was then overlapped with a list of known phenotypical features associated with mutations in a given gene. The overlapping HPO terms were used to calculate a gene sum score to rank potentially relevant genes. Such score was derived using the information content of an HPO term, which is defined as the inverse of the probability of observing the phenotype in a given database. In blue, raw data from 10x Genomics linked-reads and Bionano optical maps were processed using default settings. Variants were called and filtered based on allele frequency and the pre-defined gene list. All rare SVs were vetted against regions known to be associated with deletion or duplication syndromes. All translocations and inversions were reported.
HPO: human phenotype ontology, EHR: electronic health record, VCF: variant call format.

Supplementary Table 1. Pipeline performance.

Case	Variant type	Diagnostic variant rank
0103	Indel	1 out of 4 in de novo
0203	SNV	2 out of 10 in de novo
0503	SNV	1 out of 4 in de novo
0703	SV - translocation	1 out of 1 in translocation
0803	Indel	10 out of 26 in de novo
1003	Indel/SNV	3 out of 3 in compound het
1503	Indel/SNV	1 and 4 out of 8 in homozygous
1703	SV - duplication	1 out of 19 in SV syndromes
1903	Indel/SNV	5 out of 14 in de novo & 1 out of 5 in recessive
2103	SNV/SNV	1 out of 4 in recessive & 10 out of 13 in de novo
2303	SV - duplication	4 out of 11 in SV syndromes
2403	Indel/SNV	1 out of 8 in compound het
2704	SNV	1 out of 3 in compound het
3103	SNV	1 out of 12 in de novo
3603	SNV/SNV	1 out of 1 in recessive
4103	Indel	1 out of 15 in de novo
4203	SV - deletion	1 out of 1 in deletion
4803	SV- deletion	4 out of 8 in deletion
4903	SNV	3 out of 92 in inherited het
5103	SV - deletion	1 out of 1 in deletion

Supplementary Table 2. Additional thirty cases tested with FGA and candidates identified.

Patient	Sex	Phenotype	Event	Candidates	Prior exome	Prior array
**0603**	F	Long eyelashes, synophrys, cirrhosis, portal hypertension, pulmonary arterial hypertension	SNV	*CFLAR*, hom, c.343C>T, p.Leu115Phe	+	+
**1203**	F	Cleft lip and palate, no lip pit	SV deletion 0.53 Mb	Het deletion, paternally inh, chr4:8,971,896-9,505,960 (4p16.1), also in affected sib	+	-
**2503**	M	Hyper-IgE recurrent infection syndrome (147060)<br><br>Seizures, GDD, vasculitis, uveitis, arthritis, neutropenia†	SNV<br><br>SV deletion 15.9 kb	*STAT3**, het, *de novo*, c.2144C>T,p.Pro715Leu<br><br>Deletion, inh, Breakpoints chrX: 155532641 – 155548566, disrupts *TMLHE*	+	-
**4703**	M	Intellectual disability, prominent/narrow forehead, hypotonia, tall stature, dysmorphic, overweight, abnormal eating behavior	SV deletion 28.9 kb	Deletion, inh, breakpoints chrX:103,553,084-103,582,019, disrupts *TCEAL4* & overlaps region MRXSCS (OMIM #300861)	+	+
**5203**	M	Pulmonary arterial hypertension, hypospadia, hydronephrosis, frontal bossing, congenital nephrotic syndrome	SV deletion 11 kb	*De novo* deletion, breakpoints chr2: 218217254- 218228733 disrupts *ARPC2*	+	+
**4503**	M	Epileptic encephalopathy, situs inversus totalis, scoliosis	SNV	*SOX17, de novo*, c.415C>T, p.Pro139Ser	+	+
**0303**	F	Seizures, eyelid myoclonus, ataxia, developmental regression, abnormal brain morphology, father affected	SNV<br><br>SNV	*MFN2**, het, paternally inh, c.1252C>T, p.Arg418Ter<br><br>*GABRA1**, het, paternally inh, c.1297A>G, p.Ile433Val	+	+
**4603**	M	GDD, dysmorphic, multiple basal ganglia strokes, microcephaly, synophrys, epicanthus	SNV<br><br>Unbalanced insertional translocation<br><br>Aneuploidy	*MT-ATP8*, *de novo* m.65T>C, p.Leu22Pro<br><br>der(2)t(2;7)(q37.3; q11.23)dup(7)(q11.23;q11.23), maternally inh<br><br>XYY syndrome*	-	+

**2203**	F	Wide mouth, facial asymmetry, profound sensorineural hearing loss	SV duplication 400 kb<br><br>SNVs	*De novo* dup chrX:123,800.000-124,200,000 (Xq25)<br><br>*TECTA*, het maternally inh, c.3107G>A, p.Cys1036Tyr<br><br>*PDE1C*, het paternally inh, c.1741G>A, p.Trp581Arg	-	+
**1603**	F	Glutaric aciduria	Indel<br><br>SV duplication 100kb	*ETFDH**, het, c.1773_1774del, p.Cys592Ter, second variant not found<br><br>*ECHDC1* dup, paternally inh, chr6:127334847-127434780	-	-
**0403**	M	Cleft palate, cryptorchidism, optic nerve coloboma, hypotonia, cerebellar vermis hypoplasia, polymicrogyria, dysplastic corpus callosum, colpocephaly	SNV	*SPTAN1*, *de novo* het, c.3604G>A, p.Val1202Met	-	+
**4303**	M	Sickle Cell Anemia (603903)<br><br>Optic atrophy	Indel/SNV<br><br>SNV<br><br>SNV	*HBB*,* comp het, c.27dupG:p.Ser10fs & c.20A>T,p.Glu7Val<br><br>*VAX1,* het, maternally inh, c.103G>A, p.Ala35Thr<br><br>*DSPP de novo*, c.3742_3743insTAGCAGTGACAGCAGCT, p.Asn1248IlefsTer72	-	-
**4403**	F	Sickle Cell Anemia (603903)<br><br>Episode of hyperammonemia	SNV<br><br>SV inversion 1057 bp	*HBB**, hom, c.20A>T, p.Glu7Val<br><br>*De novo* inversion*,* breakpoints chr10:63198053-63199109, disrupts *JMJD1C*	-	+
**3503**	M	Bilateral retinal detachment, exudative vitreoretinopathy, retinal dysplasia	SV inversion 88.4 kb	*De novo* inversion, breakpoints chr16:484,033-572,449, disrupts *PIGQ* & *RAB11FIP3*	-	-
**2903**	M	Cataract, GDD, medulloblastoma, osteochondroma, hearing impairment, disproportionate short-trunk	SV inversion 10.3 kb	*De novo* inversion, breakpoints chr14:21,257,814-21,268,130, disrupts *HNRNPC*	-	+

**4003**	F	Constipation, gastrointestinal dysmotility, intestinal pseudo-obstruction	SV inversion 70.1 kb	De novo inversion, breakpoints chr16:2546651 – 2616774, disrupts *PDPK1*	-	+
**3403**	M	Speech and language delay, hypotonia, coarse facial features, hypotonia, thorax asymmetry, juvenile rheumatoid arthritis	SV duplication 1.5Mb<br><br>SV duplication 250 kb<br><br>SNV	*De novo* dup* chr1:35,213,750-36,754,000* (1p34.3)<br><br>Maternally inh dup* chr12:55,564,531-55,764,531 (12q13.2)<br><br>*L1CAM*, hemizygous, maternally inh, c.860G>A, p.Arg287His	-	+
**3803**	F	Ketotic hypoglycemia, seizures, hypermobility, chronic diarrhea, bruising susceptibility, recurrent infections	SNV	*ACADM**, het, paternally inh, c.985A>G, p.Lys329Gln, second variant not found	-	+
**1403**	M	Seizures, ataxia, leukoencephalopathy, hemophagocytic lymphohistiocytosis			+	+
**2603**	M	Cleft lip and palate, autism, pituitary dwarfism, GDD, hyperlipidemia, hypercholesterolemia			+	+
**3003**	F	Ketosis, stroke, myoglobinuria, lagopthalmos, esotropia, nystagmus elevated hepatic enzymes			+	+
**3203**	F	Decreased T-cell count, prematurity			+	+
**3303**	M	Polyneuropathy, areflexia, muscle weakness, orthostatic tachycardia, polyneuropathy			+	+
**3703**	M	Seizures, GDD, prominent nasal bridge, simple ear pinna, epicanthus inversus			+	+
**3903**	F	GDD, butterfly vertebrae, microcephaly, seizures, failure to thrive, low posterior hairline			+	+
**2003**	F	Congenital hepatic fibrosis, cholestasis, abnormal coagulation cascade, hirsutism			+	+
**0904**	M	Autism, DD, hypospadias			-	+
**1303**	M	Panhypopituitarism, schizencephaly, seizures, septo-optic dysplasia			-	+
**2803**	F	TAPVR, abnormality of the Eustachian tube, 2-3 toe syndactyly			-	+
**5303**	M	GDD, seizures, brachycephaly, wide nasal bridge, broad nasal tip, kyphoscoliosis			-	+

*also found previously; TAPVR = total anomalous pulmonary venous return; GDD=global developmental delay; hom=homozygous; het=heterozygous; dup=duplication; inh=inherited, + yes, - no.

Supplementary Table 3. Comparison of inter-chromosomal events between short-read WGS CNV and genome assembly technologies for proband 0703 with a 46,XY,t(1;9)(p32.3;p21) rearrangement.

	**Short-read WGS CNV**	**Linked-reads**	***De novo* assembly**
**Variant calls:**			
Inter-chromosomal events	729	96	18
Filtered inter-chromosomal events	496	0	4
**Diagnostic variant:**			
Identified	yes	yes	yes
Correct SV type	n/a	n/a	yes
Correct zygosity	yes	yes	no

Short-read WGS CNV = Manta output; Linked-reads = 10x genomics output; *de novo* assembly = Bionano optical mapping output.

Supplementary Table 4. Comparison of inter-chromosomal events between short-read WGS CNV and genome assembly technologies for a proband 4603 with a complex rearrangement. Duplication of 7q11.23, subsequently translocated to 2q37.3 in an inverted orientation [der(2)t(2;7)(q37.3; q11.23)dup(7)(q11.23;q11.23)]

	**Short-read WGS CNV**	**Linked-reads**	***De novo* assembly**
**Variant calls:**			
Inter-chromosomal events	427	59	19
Filtered inter-chromosomal events	305	2	2
**Translocation identified:**			
Identified	yes	yes	yes
Correct SV type	n/a	n/a	yes
Correct zygosity	yes	yes	no
**Duplication identified:**			
Identified	no	yes	*
Correct SV type	n/a	yes	*
Correct zygosity	n/a	yes	*

*Rearrangement fully identified by de novo assembly (see also Figure S3)

Supplementary Table 5. Comparison of deletion calls between short-read WGS CNV and genome assembly technologies. Table includes calls for proband 5103 with 36 kb *TANGO2* biallelic deletion.

	**Short-read WGS CNV**	**Linked-reads**	***De novo* assembly**
**Variant calls:**			
Total number	4510	4858	8644
High quality	3760	4712	1697
Mean size ± SE (bp)	40,1248 ± 14,166	53,827 ± 13,779	3,158 ± 671
**Diagnostic variant:**			
Identified	yes	yes	yes
Correct SV type	yes	yes	yes
Correct zygosity	no	yes	no

Supplementary Table 6. Comparison of deletion calls between short-read WGS CNV caller and genome assembly technologies. Table includes calls for proband 4203 with 1480 bp *WAC* deletion.

	**Short-read WGS CNV**	**Linked-reads**	***De novo* assembly**
**Variant calls:**			
Total number	4629	4649	8329
High quality	3895	4490	1666
Mean size ± SE (bp)	66,120 ± 20,869	88,137 ± 20,559	6,146 ± 1,185
**Diagnostic variant:**			
Identified	yes	yes	yes
Correct SV type	yes	yes	yes
Correct zygosity	yes	yes	yes

Supplementary Table 7. Comparison of deletion calls between short-read WGS CNV caller and genome assembly technologies. Table includes calls for proband 4803 with 5000 bp deletion disrupting *USP34*.

	**Short-read WGS CNV**	**Linked-reads**	***De novo* assembly**
**Variant calls:**			
Total number	4705	4805	9313
High quality	3911	4650	1637
Mean size ± SE (bp)	68,216 ± 20,478	42,572 ± 15,581	5740 ± 1213
**Diagnostic variant:**			
Identified	yes	yes	yes
Correct SV type	yes	yes	yes
Correct zygosity	yes	yes	yes
